# Entangled local biologies: genetic risk, bodies and inequities in Brazilian cancer genetics

**DOI:** 10.1080/13648470.2017.1326756

**Published:** 2017-07-19

**Authors:** Sahra Gibbon

**Affiliations:** ^a^ Department of Anthropology, University College London, London, United Kingdom

**Keywords:** Genetics, Brazil, cancer, local biologies, inequalities

## Abstract

Engaging recent social science work examining the truth making claims of science and biomedicine, this paper explores how biology is being localised in Brazilian cancer genetics. It draws from ethnographic fieldwork in urban regions of southern Brazil working with and alongside patients, families and practitioners in cancer genetic clinics. It examines how different sorts of ‘local biologies’ are articulated in the context of research, clinical practice and among implicated patient communities and the way these can ‘recursively’ move across different spheres and scales of social action to extend and transform the meaning of the biological. It shows how the mattering of the biological in Brazilian cancer genetics is fundamentally informed by questions of inequity and care, even while multiple local biologies may obscure rather than reveal the biopolitics of cancer. In an era of epigenetics this raises new opportunities and challenges for anthropological analysis as intervention.

The way that the truth making claims of science, including those related to genomics, are reproduced and sustained, has been widely examined by social scientists drawing on a range of theoretical approaches from medical anthropology and Science and Technology Studies (STS)(Taussig 2009; Rapp 2000; Fullwiley 2011; Reardon 2004; Gibbon et al. 2014). From these perspectives genes, genetic knowledge and technologies are ‘co-produced’ as an ‘assemblage’ of social and biomedical discourses and practices. As the same time efforts to move beyond the ‘body proper’ (Lock and Farquhar 2007), to ‘trouble, problematise and scrutinise natural categories’ (Lock 2013) and challenge notions of a universal biology reflect long standing interest in medical anthropology with what have been described as ‘local biologies’. Informed by Margaret Lock's work on menopause in Japan this concept has been used to illuminate the contingency and interdependency of the material and the social and underline the need to examine the embodied experience of health and illness in specific local contexts, the cultures of biomedicine and the ways that the biological and social are increasingly entangled (Lock 2001; Lock and Nguyen 2010). It is a concept which has gained renewed force and relevance in a post-genomic era in which the biological seems to be increasingly localised from the ‘ inside out’, with growing uncertainty about the stability of ‘the gene’ and the complexity of gene function vis-a-vis variously defined environments (Lock 2013). It is a central tool in this special issue in considering the challenges, opportunities and limits of new theoretical approaches for making visible, engaging and understanding the significance of diverse bodies and biologies.

This paper draws from these different approaches to examining the (changing) ontological status of the biological to illustrate how a variety of local biologies are articulated, manifested and intersect in cancer genetic research and medical practice in southern Brazil. It is based on 18 months ethnographic fieldwork working with and alongside patients, families, scientists and practitioners in three urban cancer genetic clinics.[Fn en0001] It outlines how in an era of transnational globalisation of genomics the localisation of the biological in Brazilian cancer genetics is taking place across different domains of social action and how this involves a variety of different assemblages that displaces some bodies, biologies or materialities, whilst cohering the interconnections between others. It reveals the mattering of the biological in Brazilian cancer genetics to be fundamentally informed by questions of inequity and care. At the same time these multiple but ‘less than many’ (Mol 2014, 2003) biologies reflect not only difference or disjuncture but cross cutting, clashing and sometimes generative intersections. In this way the paper illuminates how cancer genetics in Brazil is not only shaped by an engagement with and articulation of diverse local biologies but how these move across different spheres and scales of social action to both extend and/or transform the meaning of the biological. In examining these shifting and in some cases ‘recursive’ dynamics, the paper engages with work in anthropology examining how scientific ‘facts’ associated with reproductive and genomic technologies are subject to ‘domaining’ (Strathern 1992) or ‘analogic return’ (Franklin 2014), as well as Hacking's notion of ‘looping effects’ (2006). The paper outlines how this entanglement of local biologies operates within and at the intersection of three different arenas of social practice in Brazilian cancer genetics. This includes scientific research agendas in which genetic ancestry and cancer as an increasingly stratified disease is foregrounded in pursuit of yet-to-be known cancer risk; the precarious health infrastructure of Brazilian cancer genetics where the boundary between care and research is thin and marked by inequities as well as limited resources and finally the embodied experience of cancer risk in which socio-historic specific articulations of bodily vulnerability are being contemporarily reconfigured.

## Cancer genetics in Brazil: unknown risk and research in pursuit of local biology

Brazilian cancer genetics emerged in early 2000s at the meeting points between a growing public health concern with rising rates of cancer incidence and mortality and national and transnational research agendas which constitute cancer as an increasingly differentiated disease. It is an arena of scientific research and medical practice in Brazil which is unfolding within a domain of severe resource limitations, with consequences for the inter-relationship between research and care.

Particularly high recorded rates (equivalent to the US population rates) of breast and prostate cancer, across the southern regions of Brazil, have long been noted by the National Cancer Institute (INCA 2009). Constituted as part of the effort to address what is a growing public health concern, the hub of an emerging field of cancer genetics has developed linked to large, mostly public health or university hospital research institutes in these same regions. Brazilian cancer genetics is not however currently integrated into the public health care system or *SUS* and is almost entirely reliant on research funds and collaborations. The often fluid boundary between clinical care and research objectives is a feature of the ‘bio-clinical collectives’ (Bourret 2005) that characterise cancer genetics more generally (see also Hallowell et al. 2009). Nevertheless this has specific consequences when public health services are fractured and uneven as they are in Brazil. As a result patients and families are recruited into programmes of care via research protocols, linked to high profile areas of inquiry such as BRCA genes or research on ‘rare’ cancer syndromes. Eligibility for participation in research (and the care this promises) are therefore highly contingent on the availability of research funds. This can leave many patients and families waiting for months or sometimes years for a conclusive test result. They are as a result stratified ‘patients-in-waiting’ (Timmermans and Buchbinder 2010), dependent on the availability of resources to pursue genetic testing via research or sometimes left not knowing the clinical relevance of an identified biomarker or mutation. As I examine in the latter part of the paper, this is a terrain of uncertainty which has particular consequences for how patients understand and engage with the ontologies of embodied genetic risk.

Taussig et al. illuminate how the potential of the not-yet-known propels many novel fields of development in the life and medical sciences (2013). This includes Brazilian cancer genetic research as it unfolds in dialogue with international research in which cancer is being increasingly biologically diversified and stratified and as new genetic variants and other biomarkers become identified as potential targets for the promise of personalised medicine (Lee 2013). This has enabled as yet ‘unknown’ genetic risk of cancer to be defined as an object of research in Brazil where a particular focus on genetic ancestry and population difference is foregrounded in efforts to address and intervene on rising yet regionally differentiated national cancer rates. It is a focus which is reflected in the National Brazilian Hereditary Cancer Network's stated goal of ‘needing to know and characterise the particular aspects of our population’ (INCA 2009), providing a further illustration of how genetic risk of cancer in Brazil is situated in terms of a local biology.

Nevertheless the research focus on genetic ancestry is characterised by a dual emphasis in Brazilian cancer genetics which produces mutable and mobile biologies. On the one hand there is an emphasis in scientific publications on regional specificity associated with migratory histories, particularly the influx of European migrants to the southern regions of Brazil in the early nineteenth and twentieth centuries. This has justified an initial investment in research focused on identifying the frequency of common well-characterised BRCA founder mutations. On the other hand there is an emphasis on national ‘tri-hybrid’ ancestries, that also demarcate the region as different from the US and European context (see Gibbon 2016a). This movement between alternately highlighting ‘heterogeneity and homogeneity’ has been widely noted by other social scientists examining how population genomics in Brazil and the wider region of Latin America is informed by and configures particular ideas of nationhood, identity and race (Wade et al. 2014; Kent, Santos and Wade 2014). Santos et al. suggest that a focus on genetic ancestry in Brazilian pharmacogenomics research has served to underlie the specificity of both Brazil and the Latin American region (2015). In a similar way the foregrounding of genetic ancestry in Brazilian cancer genetics works to articulate the importance of the local biologies that might be contributing to rising and variable cancer incidence. These are configured however not only in terms of ‘unknown’ risk but also in relation to ‘under-served’ populations (Gibbon 2015); an emphasis which also reflects wider dynamics in the way the globalisation of genomics is being tethered to questions of social justice (Fullwiley and Gibbon forthcoming).

While inequities are highlighted within the research aims of Brazilian cancer genetic research, the day to day reality of limited health care resources also directly shape clinical practices. Stratified abilities to leverage and access medical interventions inform the ‘ontological choreography’ (Thompson 2005) of clinical practice in Brazilian cancer genetics. In the clinic particular kinds of risk and bodies are mattered or made material with consequences for the local biologies made possible and reproduced at this interface.

## Materialising risk and care in a context of public health inequities: abject and distributed bodies

An ethnographic excerpt from my field notes illuminates how certain materialities, in this case living with cancer and the stark reality of a lack in public health resources, disrupt and disturb the preventative promise of cancer genetics.

In a busy morning in the cancer hospital in Rio the normal long queues are forming in the large waiting room area, where oncology and cancer genetic patients are waiting to be seen, next to the cramped consulting room area that the cancer geneticists and oncologists must negotiate between appointments. Many cancer patients are identifiable in the waiting areas clustered around the inadequate seating together with their families. With drips attached to their arms they are characterised by pale anxious faces and often hairless heads. Several are clutching notes in plastic bags, or large thick battered folders from which x-ray scans and other medical images are visible.[Fn en0002] Cancer patients, while noticeable in the hospital grounds, entrance and waiting areas, were however mostly absent from the cancer genetic clinics. For the most part patients in these clinics are relatives of those who have had or were being treated for cancer. However at the end of the morning clinic in Rio a couple in their late fifties squeeze themselves into the small consulting area. The man has a drip entering his nose and he is pale, thin with a noticeably grey pallor, made even more evident by an absence of hair and eyebrows characteristic of those being treated for cancer. The woman explains that the consultant they have been seeing in the hospital referred him to the cancer genetic clinic because of the family history. It is clear that the geneticist is uncomfortable and reluctant to fully investigate these aspects when the mostly silent man is clearly so unwell and in the middle of treatment. The clinician discovers that he is about to go upstairs for a session of radiotherapy, although the woman states that they are ‘not sure if the equipment is working today’. After a fleeting discussion of the history of cancer in the family the conversation turns to the treatment protocol the patient is receiving, with the geneticist asking who is overseeing the husband's care in the hospital. This prompts the woman to talk about the long and costly journeys they frequently have to make from the outskirts of the city on public transport to get to the hospital, because there is currently no reliable hospital transport service. It is a short consultation with the normal focus on who is at risk in the family and who could be entered into a cancer genetic research protocol or be eligible as a result for genetic testing displaced by the everyday reality of managing cancer treatment and lived bodily manifestation of its consequences. When the couple exit the geneticist is exasperated at the referral, railing at the ignorance of some of the oncology team in sending a cancer patient who is clearly not well to the genetic clinic at this stage in their treatment.

The presence of what might be seen as the ‘abject’ cancer body in this ethnographic illustration serves to inadvertently expose for both the geneticist and patients the shortcomings of Brazilian cancer genetics in a context of inadequate health care provision. The prospect of participation in research that may take months if not years to yield a result of questionable utility is subsumed by the challenges of treatment and care for the cancer patient. It suggests that the lived and embodied reality of cancer is not easily managed within the space and practice of Brazilian cancer genetics and is sometimes revealed as inappropriate care. Such inequities also however have consequences for how the biological in the cancer genetic clinic is mattered through an assemblage of ‘hidden’ technologies which work to articulate a local biology in which distributed genetic risk is emphasised.

Social scientists have noted how the practices of the cancer genetic clinics in diverse locales are widely characterised by attention to not only the health or future cancer risk of the patient attending the clinic but the past, present and predicted risk of other relatives and/or future generations (Hallowell 1999; Gibbon 2007). Often the person in the clinic seeking information does not have cancer themselves or may discover that they may not be at most genetic risk of the disease now or in the future. This is visibly evident in the way that risk assessment is calibrated most frequently on the basis of family history made material and present in the collective attention to the medical family tree (see Gibbon 2002). This is also a significant dimension of the practices of clinical cancer genetics in Brazil which serves to constitute a relational patienthood that situates risk on the basis of past histories of cancer in the family and for future generations. As a result, despite painful memories of cancer often ‘haunting’ clinical consultations, the ‘lived cancer body’ is frequently displaced or made somewhat absent, as the above ethnographic excerpt illustrates. Moreover a dependency on research funds and the frequent lack, due to a shortfall in resources, of routine access to genetic testing make efforts to draw up detailed clinical family trees the substantive component of clinical routines and practices, further foregrounding the relational aspects of genetic risk.

While the family tree is one key dimension of Brazilian cancer genetics other materialites are also central. These not only serve to similarly emphasise the relational aspects of cancer risk but also reflect how inequities in health care shape which bodies have the potential to matter.

In the mixed public/private hospital in Sao Paulo patient and family case notes would be visibly marked on the front stamped in red letters *SUS* or *CONVENIO* to inform the practitioner if the patient was a public health patient or if they were part of a private health insurance scheme. Notes stamped with *convenio* would travel to and from the consulting space as administrators and practitioners attempted to negotiate with private insurance providers to establish who within the family would be eligible for or who could seek additional screening. Whilst securing a genetic test in this way was unlikely,[Fn en0003] additional screening and monitoring was often possible via negotiations with private insurance schemes, not only for the individual patient with a *convenio* but also sometimes for other related family members. As patients and extended family members waited in the consulting room there would be hasty telephone calls by the clinic's administrator to establish who and what could be covered by the insurance provider; an outcome which would often inform final decisions about treatment programmes and screening interventions. With many patients commenting upon their ‘luck’ at being seen within a leading cancer research hospital where according to one patient, ‘everything is a blessing’, being able to access extra screening services for oneself and one's family in the context of a fractured system of public health care was seen as an added benefit. In a similar way that producing the clinical family tree distributes cancer risk beyond any individual body, negotiations related to *convenios*, concerning access to screening and care services, have an equally displacing affect. As a result attention is focused not only on traumatic personal histories of cancer but also on relations as social obligations and rights, between family members.

Collectively these practices constitute a key part of the clinical routines of Brazilian cancer genetics, materialising bodies and relational risk at the interface with the financial and resource limits of research and care in specific ways. The contingencies which are quite literally mattered in clinical practice centre on the family tree and collective efforts to gain access through research to genetic testing or rights to basic care such as mammography screening on the basis of having a *convenio*. These practices co-produce a relational local biology which is directly shaped by the reality of health care inequities. However for patients cancer risk is made meaningful and present in ways that foreground other bodily and non bodily materialities, evoking seemingly different local biologies.

## Embodying cancer risk: emotion and social relations in the family

For many patients attending cancer genetic clinics in Brazil, genetic mutations were rarely understood as the sole or sufficient cause of cancer, but were almost always interacting with other factors. Sometimes a generalised notion of stress, would be implicated. This was for instance the way that a female middle aged patient from Porto Alegre talked of how daily stress might enter and act on the body:

‘because you are in that state of ‘*pique*’ [meaning alertness, anxiety or stress] all day and the body seems like its not being affected you know but you go about really hyped up and your cells really hyped up or your blood.. and while you are like this your body is tired, your head is buzzing and thinking, thinking things it shouldn't, so I think a lot of it is stress for breast cancer – *stress is a something that makes your antibodies slow*. [my emphasis]

However many patients went beyond the discussion of a generalised notion of stress, talking specifically about the way that ‘negative’ emotions acted on the body in producing a risk for and/or causing cancer. Emotions were something that could enter and co-produce bodies and disease. In part this reflects the growing relevance in a contemporary Brazilian context of a psychologically infused notion of the self or ‘psi-self’ (Duarte 2000). But it also is an expression of an understanding of bodies as porous, co-produced as a consequence of a problematic past or lived relations in the family as well as the intergenerational consequences of suffering or trauma. As the case studies outlined below illustrate *emoções* have an agentive and mattering consequence on bodies in ways that link the trauma of familial cancer to poverty, violence, religion, pollution and diet as well as reflecting a high profile media discourse about psychological self-management.

Ana Paula was in her early 40s, she lived in Porto Alegre and worked in a shoe factory in one of the urban suburbs of the city. As well as having had breast cancer herself, a very large number of her family had had cancer including her father, a number of sisters and most recently her teenage daughter who was currently being treated for a rare bone cancer. The family were under the care of the *oncogenética* team in the hospital and were waiting the results of blood tests to confirm if a genetic mutation had been identified in the family. During our first meeting Ana Paula recounted in detail these traumatic experiences of cancer as she had grown up. She recalled the physical horror of those relatives who had had debilitating experiences of suffering and in many cases dying of cancer, pointing out how this had understandably ‘marked’ her greatly. She told me that since being treated at the public hospital she had always heard that it ‘could be genetic’ adding ‘it could be that different bolts of lightning fell in the same place at the same time’. In fact elaborating further later in the interview she told me that she always thought that it was ‘emotional’. This was how she put it:

‘I am always hearing interviews on the television with doctors about where breast cancer comes from? It comes from continuous hurt, from anger I'm always hearing this. All the time I was having treatment I heard this that it comes from genetic inheritance so it could be emotional factors ..and in my family too I'm beginning to think that it's this.. to be certain that it's the two together because our head co-ordinates our body. I think that feelings are part of our daily existence and you don't know but one day you say something bad and you hurt someone or you are hurt. These are things that you can't predict. I think that it's this because my husband left me when I was unwell and then I started to hold my sadness, my hurt and then the breast cancer developed. I did all my treatment alone and finally my cancer was sleeping. But I was deceived by someone else I was living with and the cancer returned in another place’.

For Ana Paula genetic inheritance is inseparable from the inter-relational and embodied effect of emotions on the body and the self from others. At the same time this seems in part also informed by the clinical information she has been given about genetic risk and a media discourse about psychological well-being.

Luiza Maria expressed similar sentiments about the cause of cancer as related to her family relationships. She was 45 years old and worked as a legal secretary in Sao Paulo and had been treated for breast cancer twice first in her early 30s and again more recently. There had been other cases of cancer in her distant family and she was awaiting the result of a genetic test on the BRCA genes. She talked openly about how she had found the surgery and treatment she had received as ‘mutilating’ and how this had caused psychological problems in her marriage of ten years. This is what she said in response to a question about what she thought the ‘causes’ of cancer were.

I think cancer can occur because of emotional problems. I lost a loved one in the house that I was building in an accident and this really affected me [*isso me marcou muito*]. I am a person who somatises [using the verb *somatizar*] problems. Unfortunately, I'm very connected to family. I had a brother who at the same time got involved in drugs, so I was very sad about all this. Soon after my husband lost his job, my father died all very close together. So I think that it's all because of this, because I'm a very emotional person, because I always go behind problems, I worry about them too much. All this ‘somatisation’, I think it just left me really sad without a way to resolve my problems.

Liliane was in her late 50s and had now retired after being a teacher for many years. She had had a traumatic and troubling history of cancer in the family which she recounted in great detail at the start of our meeting telling me how her daughter, sister and nieces had all had cancer. She herself had a few years ago undergone treatment for colon cancer and like other members of the family had been identified as a carrier of a particular founder mutation R337, linked in Brazil to an inherited cancer syndrome known as Li-fraumeni. While she acknowledged this as a factor, like other patients, she also foregrounded the agentive consequences of emotional dynamics in the family. As the exchanges outlined below illustrate for Lilliane the meaning of ‘predisposition’ was a complex mesh of social and psychological factors.

Liliane: Like she said [the geneticist] we have the predisposition. Mine in particular, not like my daughter who was a baby and came with the genetic factors and perhaps others in the family too. But this niece that died of adrenocortical cancer, she had a problem in the house, she lived with conflict even when she had the disease. And my other niece who had breast cancer she had a problem too as a baby. My sister married, she had a child, but the father was an alcoholic,… so my sister really had a history and I think my niece suffered too. Later she remarried and they had a good life, he was like a father to me in fact, but he died and it was like losing my father.. So I think that I have a history.

Sahra: Do you think that this history is more important than other risk factors for cancer?

Liliane: I think that it interferes (*mexe*).. messes with a person. I think that in the end it left something inside. But in that period when I discovered the cancer, nearly 2 years ago..I was experiencing strong emotional problems. I was hurt (*estava magoada*).. so when I discovered I was very sad, things started happening which messed with me (*mexendo conmigo*). Even when I went to the doctor and he said to me ‘You don't have any reason to have this, a healthy person, you don't smoke, you don't drink, you exercise.’ He even joked ‘what frog did you swallow’ ? (*Que sapo voce engoliu)* [Fn en0004]

So I think this increases the chance a little more. Given what we are hearing people say, what we read on the internet, at times you read in a book. I've read books that say emotional aspects are everything. Emotions interfere with the entire body I think.

The emphasis on the embodied and mattering consequences of suffering, hurt, sadness and negative emotions in these narratives refute the notion that for these patients genetic mutations provide the necessary explanatory parameters for cancer in the family. Significantly even when genetic risk is concretised, as it had been for Lilliane following a positive test result, there is still an emphasis on the interaction between the biological and particular forms of sociality and social relations in rendering meaningful cancer as an illness experience or in terms of its embodied danger.

## Entangled local biologies

These apparently ‘de-molecularised’ readings of cancer and bodily risk (see Gibbon 2016b) resonate with well recognised ‘folk’ understandings of the body in Brazil and Latin America where, as Roberts points out, the ‘reciprocal malleability of bodies and environments’ has long been evident (2015). Social science work in Brazil illustrates the variety of ways the sick body is often perceived as being subject to and produced through exogenous influences, including illnesses such as *nervismo* in Brazil (Duarte 1986) or the embodied consequences of strong emotions for health (Rebhun 1994).[Fn en0005] Recent research highlights how ideas about porous bodies also resonate in the context of biomedical interventions such as IVF, hormones and cosmetic surgery in different regions of South America (Roberts 2010; Edmonds 2011; see also Sanabria and Lowy 2016; see also Rohden 2001). Emilia Sanabria's work examining hormonal menstrual suppression in Bahia in the north east of Brazil demonstrates how humourally inflected notions of blood are an expression of the body not as fixed or defined by rigid boundaries but as contingent and subject to flow and transmutability (2016). But as the work of Sanabria (2016) and Edmonds (2011) illuminates, bodily plasticity in the context of hormone treatment or cosmetic surgery finds expression in Brazil in dialogue with contemporary biomedical interventions, not outside or beyond them.

In a similar way I would argue biomedical discourses about genetic risk and patients’ rendering of embodied risk becomes part of an expression of diverse local biologies in which cancer, emotions and the vulnerable body are co-constituted. Clinical dynamics aimed at identifying high risk families are often intensely focused with medical family history or with negotiating collective, yet ultimately differentiated, access to care in ways that necessarily shape past understanding of and contemporary lived social relations in the family. As histories of cancer in the family are re-lived and familial rights and obligations aligned in the negotiations surrounding access to care, testing and screening, a certain licence is afforded to these broader understandings of the mutable body as informed by emotional relations between kin. Notable here is Ana Paula's response that the cause of her cancer is ‘emotional’ after hearing in the clinic that her cancer could be linked to genetic factors or the way that Luiza Maria equates herself as a person who ‘somatises’ illness because she is ‘unfortunately connected to family’. Similarly, Lilliane's understanding of ‘pre-disposition’ reflects difficult family relations and stands in contrast to other affected relatives, such as the children with cancer in her family, who only had a ‘genetic’ risk. These illustrative examples suggest that between clinical communication and patients’ embodied understanding of genetic risk there is a ‘looping effect’ (Hacking 2006) or ‘recursive return’ (Franklin 2014). For the patients I met this re-inforces the bodily relevance of problematic emotional difficulties in interpersonal relations which are then read back onto the significance of potential genetic risk that is emphasised by clinicians. Importantly whilst this movement coheres the reality of genetic risk for patients, for many practitioners its presence represented something of a disjuncture or a ‘clash’. For example, a number suggested, that the agency given to negative emotions by patients erroneously sustained familial blame and guilt, instead of seeing cancer in the family as ‘nobody's fault’, as one doctor put it.

A further illustration of the ‘looping’ intersections between different localisations of the biological in Brazilian cancer genetics is also made evident in examining how the relevance of genetic ancestry was sometimes incorporated and woven through patients’ understanding of embodied risk for cancer. While discussions about genetic ancestry were most explicit in the context of international research publications and collaborations, my research suggests they also find defuse and subtle articulation within the clinical contexts and in interaction with patients (Gibbon 2016a). This can further entangle different local biologies as patients configure biomedical information about cancer risk and ancestry alongside a sense of embodied vulnerability.

An explicit discussion of genetic ancestry was not frequent in the clinical consultations I observed. More usual were implicit statements by a clinical geneticist about the fact that an identified genetic mutation might be ‘more common in the south’. Sometimes this would be associated with the history of migration to certain parts of Brazil, particularly the arrival of the Portuguese or European populations in the nineteenth and twentieth centuries. These fleeting comments could however precipitate a more reflective discussion from patients and their relatives about regional identity or family origins/ancestry. As the case study outlined below illuminates an awareness of the relevance of genetic ancestry to clinical research might not only confirm cancer risk as relationally constituted across generations, but serve to evoke the wider context of gendered roles in the family and the agentive role of emotional suffering.

Marcia was in her early fifties and worked as a paediatric nurse in a public hospital in Porto Alegre. She had had cancer a number of years previously and was told by the geneticist that given the number of cases of breast and ovarian cancer in the family that there was a high risk of identifying a genetic mutation in the family. She and other members of her family despite being involved in research protocols for a number of years still had not received confirmation that a deleterious mutation had been identified. Marcia did not hesitate to tell me about how she saw the complexity of cancer. In her eloquent and thoughtful responses to my queries she made it clear that genetic factors were always in interaction with a range of other aspects of individual, gendered and more importantly collectively lived lives. She put it like this.

Cancer has various origins. There is the genetic component, environmental factors, cigarettes, alcohol, pollution, we know these things exist. But along with this I think there exists a personal aspect in some way that makes you more vulnerable to cancer in certain parts of your body. So in our family we have large numbers of women with breast cancer. Why is it always breast cancer? Of course we have the genetic factor but why always breast cancer ? All the women in my family are from large families, women who really had to struggle to keep their families, their husbands and their children. So I really think there exists this mental component that ends up in the most vulnerable organs.

In another part of our interview she mentioned this again talking about how her grandparents came from Germany during the period of widespread migration to the south and interior part of the southern state of Rio Grande do Sul and how this might contribute to cancer risk in the family. In the dialogue that followed this was further clarified.

Sahra: Do you really think that this is a risk factor for having cancer ?

Marcia: Yes with respect to the genetic question..I remember doing that tree with Dr H and we saw all those generations, so this history is present in our family. Perhaps as it passed between generations the genetic factor changed some things and of course this is related to the emotional question too.

Sahra: I'm interested to know more how you think cancer is associated with being German or a certain emotional state?

Marcia: Not only with the emotional question but the female question. The women were very subjugated, only looking after the children, big families, hard work, always giving too much, so they lived in this situation of what we could call emotional poverty. So I think this cultural question interacts, has interacted and is interacting with our genetic predisposition so that it's possible this question of German culture will mean other future generations with breast cancer..who are these women with breast cancer in the south? They are women originally from Germany, Italy, Brazilian mothers with this ancestry. But I think this question of female culture is important even though in recent decades they are more liberated we could say..in the south we say we are more advanced but its really very provincial, we are still very rigid here in certain ways.

Sahra: So you think that this cultural factor influences in a certain way?

Marcia: I think it influences Sahra in the way I already mentioned. In the moment when we are immunologically more vulnerable we develop a cancer. Even though it's been proved that if you have the gene you get breast cancer. But there are people now who work in science who are turning to the social and cultural aspects. Because these cultural and emotional questions that are provoked by our thoughts, cause chemical reactions, molecular, neurological changes inside our bodies. They translate into something concrete. Our thoughts are not just our thoughts they occur inside our body as well.

Marcia's narrative not only highlights the difficulty of disaggregating the social and the biological in the way that patients I encountered understand and make sense of cancer risk, but how specific elements of a biomedical discourse concerning genetic ancestry diffusely inform a sense of embodied vulnerability. The traumas experienced by Marcia's family in their migratory experiences indelibly marks gendered bodies and is transformed ‘into something concrete’ that is then passed on down the generations as cancer risk. While the discussion of ‘female culture’ evokes a wider politics of gender relations, this is nonetheless mostly framed by Marcia in terms of ‘emotional poverty’ where ‘thoughts’ can literally matter the biological. Importantly we also see in this example not only how clinical idioms ‘loop’ back to inform patients’ understanding of embodied risk, but how the latter move recursively forward. Marcia's reading of bodily risk and danger powerfully evokes and resonates with an emerging biomedical narrative about epigenetics in which past traumas during ‘critical windows’ of development are increasingly thought to be relevant to addressing and understanding a range of disease pathways (Panofsky and Landecker 2013). Notwithstanding Marcia's own awareness of the relevance of an emerging science of epigenetics, as herself a health professional, her comments point to another dimension of what are likely to be numerous new loops and returns in the entangled local biologies of cancer genetics, in Brazil, as elsewhere. As the interactions between genes and environments gain traction across diverse terrains of cancer research and become embedded into clinical routines it will be important to examine how different understandings of the biological and disease risk (both biomedical and so called ‘folk’ models) are mutually re-shaped.

## Conclusion

Drawing on ethnographic research in the context of clinical cancer genetics in Brazil this paper has examined how practitioners, researchers and patients engage with the biological ontologies of cancer risk. Informed by longstanding and on-going engagement with localising the body and the biological in medical anthropology, it has critically considered how particular ‘local biologies’ are reproduced across and within different social terrains encompassed by Brazilian cancer genetics. This includes research efforts to address national public health priorities through transnational collaborations; the contingencies of clinical practice and in the ways embodied risk is understood and constituted by patients and families. Beyond simply highlighting diversity the paper has illuminated how the biologies produced within these social contexts intersect, sometimes clash, but also loop back to shape a range of narrativised risk discourses and practices encompassed by and configured within Brazilian cancer genetics. It has underlined the extent to which health care inequalities are deeply implicated in the way that bodies and different articulations of the biological are being configured.

Specific kinds of local biologies are sustained in scientific research related to genetic ancestry in Brazil. This is mobilised in pursuit of national and transnational research collaborations aimed at characterising ‘unknown’ genetic risk in a context of rising and regionally variable cancer incidence, against a background of ‘under-served’ need. At the same time the biologies that are quite literally mattered in the clinical domain are directly configured by inequities in accessing care. This serves to displace some materialities, including the ‘abject’ cancer body, while bringing others into view. The foregrounding of the clinical family tree and the *convenio* in the day to day practices of the clinic are shaped by the reality of health care inequities, whilst also highlighting the relevance of a relational biology in assessing risk and pursuing care. For patients, genetic risk is made meaningful through ideas of bodily vulnerability where emotion and sentiment accumulate as risk and danger within individual bodies and also, sometimes, across generations. The way that social relations and affect are constituted by Brazilian patients as embodied danger reflects both a mutability and plasticity in how cancer risk is inferred from, within and between bodies. Yet these ‘folk’ readings of connected body-selves are also informed by clinical practice and discourse, including an implicit focus on genetic ancestry. In this way ideas about a mutable or contingent body informed by emotional vicissitudes of the self and in relation to others, often across generations, intersect and at times cohere in powerful ways with a clinical discourse about genetic risk. While the agentive role of emotions is mostly foregrounded in patients’ articulations of seemingly ‘porous’ bodies, concerns about pollution, poor diet, poverty, gendered roles or violence are also sometimes evoked. Nonetheless these local biologies work mostly to stabilise rather than challenge a biomedical narrative of genetic risk, despite on-going scientific contingency about genes and the risk they constitute vis-à-vis a range of environments. For the moment, patients’ embodied narratives do not themselves directly reveal the wider bio-politics of cancer inequities, even when as the paper demonstrates, this directly shapes the practices of Brazilian cancer genetics.

As multiple local biologies become increasingly articulated in the evolving science of cancer epigenetics across uneven and inequitable terrains of health care, in Brazil and elsewhere, it will be important to monitor how and in what ways patients’ understanding of bodily plasticity and vulnerability are informed by emerging scientific paradigms and also shape novel explanations of cancer aetiology. While biomedical narratives of epigenetic cancer risk are likely to provide a powerfully seductive explanatory frame it will nevertheless be vital to monitor the meeting points and intersections between the ensuing different, dynamic yet not *necessarily* disconnected local biologies. At stake is the extent to which epigenetics facilitates or forecloses a politicisation of health in exposing or silencing wider social and economic determinants, with consequences for how both individual and collective responsibility is formulated and acted upon (Lock 2013; Panofsky and Landecker 2013). As such attentiveness to the dynamics by which the biological is being (re)made in an era of post-genomics creates new opportunities and challenges for anthropological analysis as intervention. This is particularly when, as this paper illuminates, anthropological theorisations of the changing biological are taking place alongside and at the same time as patients, health professionals and scientists are undertaking their own theorisations.[Fn en0006] Medical anthropology with its history of challenging the idea that bodies are everywhere the same has much to contribute in ethnographically engaging and articulating the theoretical approaches that will prove vital to this task within and far beyond its sub-disciplinary focus.

## Notes

1. Research consisted of observations of clinical encounters and semi-structured interviews with patients and their families attending three different cancer genetic clinics in urban centres in the south of Brazil from 2010-2012. Interviews were also undertaken with a range of scientific and medical practitioners who were working in the clinical or research environment of cancer genetics

2. This is indicative of the fact that it is mainly patients’ responsibility in the public health hospital to store and bring these notes and files to the clinic.

3. Efforts were sometimes made to pursue a genetic test in this way but were rarely successful. During the time of my research most private health schemes, which are held by approximately 25% of the population, did not pay for genetic testing. This situation changed in 2013 when the Brazilian government passed legislation stipulating that certain genetic tests should be covered by private health insurance schemes.

4. This is a Brazilian expression meaning ‘to swallow your pride’. See Rebhun (1994) for further discussion.

5. This illness has parallels with other conditions such as *susto* Mexico (Rubel, O'Nell and Collado-Ardon 1991).

6. I am grateful to the anonymous reviewer of this paper for highlighting this dimension in their review.
